# Risk Stratification for Postoperative Acute Kidney Injury in Major Noncardiac Surgery Using Preoperative and Intraoperative Data

**DOI:** 10.1001/jamanetworkopen.2019.16921

**Published:** 2019-12-06

**Authors:** Victor J. Lei, ThaiBinh Luong, Eric Shan, Xinwei Chen, Mark D. Neuman, Nwamaka D. Eneanya, Daniel E. Polsky, Kevin G. Volpp, Lee A. Fleisher, John H. Holmes, Amol S. Navathe

**Affiliations:** 1 Department of Medical Ethics and Health Policy, University of Pennsylvania Perelman School of Medicine, Philadelphia; 2 Leonard Davis Institute of Health Economics, University of Pennsylvania Perelman School of Medicine, Philadelphia; 3 Predictive Healthcare, University of Pennsylvania Health System, Philadelphia; 4 University of Pennsylvania, Philadelphia; 5 Department of Anesthesiology and Critical Care, University of Pennsylvania Health System, Philadelphia; 6 Department of Biostatistics, Epidemiology, and Informatics, University of Pennsylvania, Philadelphia; 7 The Wharton School, University of Pennsylvania, Philadelphia; 8 Corporal Michael J. Cresencz Veterans Affairs Medical Center, Department of Veterans Affairs, Philadelphia, Pennsylvania

## Abstract

**Importance:**

Acute kidney injury (AKI) is one of the most common complications after noncardiac surgery. Yet current postoperative AKI risk stratification models have substantial limitations, such as limited use of perioperative data.

**Objective:**

To examine whether adding preoperative and intraoperative data is associated with improved prediction of noncardiac postoperative AKI.

**Design, Setting, and Participants:**

A prognostic study using logistic regression with elastic net selection, gradient boosting machine (GBM), and random forest approaches was conducted at 4 tertiary academic hospitals in the United States. A total of 42 615 hospitalized adults with serum creatinine measurements who underwent major noncardiac surgery between January 1, 2014, and April 30, 2018, were included in the study. Serum creatinine measurements from 365 days before and 7 days after surgery were used in this study.

**Main Outcomes and Measures:**

Postoperative AKI (defined by the Kidney Disease Improving Global Outcomes within 7 days after surgery) was the primary outcome. The area under the receiver operating characteristic curve (AUC) was used to assess discrimination.

**Results:**

Among 42 615 patients who underwent noncardiac surgery, the mean (SD) age was 57.9 (15.7) years, 23 943 (56.2%) were women, 27 857 (65.4%) were white, and the most frequent surgery types were orthopedic (15 718 [36.9%]), general (8808 [20.7%]), and neurologic (6564 [15.4%]). The rate of postoperative AKI was 10.1% (n = 4318). The progressive addition of clinical data improved model performance across all modeling approaches, with GBM providing the highest discrimination by AUC. In GBM models, the AUC increased from 0.712 (95% CI, 0.694-0.731) using prehospitalization variables to 0.804 (95% CI, 0.788-0.819) using preoperative variables (inclusive of prehospitalization variables) (*P* < .001 for AUC comparison). The AUC further increased to 0.817 (95% CI, 0.802-0.832) when adding intraoperative variables (*P* < .001 for comparison vs model using preoperative variables). However, the statistically significant improvements in discrimination did not appear to be clinically significant. In particular, the AKI rate among patients classified as high risk improved from 29.1% to 30.0%, a net of 15 patients were appropriately reclassified as high risk, and an additional 15 patients were appropriately reclassified as low risk.

**Conclusions and Relevance:**

The findings of the study suggest that electronic health record data may be used to accurately stratify patients at risk of perioperative AKI, but the modest improvements from adding intraoperative data should be weighed against challenges in using intraoperative data.

## Key Points

**Question:**

Is adding preoperative and intraoperative data associated with improved risk stratification of patients undergoing noncardiac surgery for postoperative acute kidney injury?

**Findings:**

In this prognostic study of 42 615 patients who underwent noncardiac surgery, the addition of preoperative to prehospitalization data improved model performance (area under the curve increased from 0.71 to 0.80) as did adding preoperative plus intraoperative data (area under the curve further increased to 0.82).

**Meaning:**

Although electronic health record data may be used to accurately stratify patients at risk of postoperative acute kidney injury, there appears to be only modest improvement in performance when adding intraoperative data to risk stratification models.

## Teaser

This prognostic study evaluates the addition of preoperative and intraoperative data to prehospitalization data to identify patients at risk for postoperative acute kidney injury after undergoing major noncardiac surgery.

## Introduction

Acute kidney injury (AKI) is a common postoperative complication, occurring in 12% of patients undergoing surgical procedures,^1^ that has been associated with poor clinical outcomes, including the development of chronic kidney disease, increased health care use, and death.^2,3^ Because of evidence describing the association of AKI with mortality,^4^ there has been heightened interest in improved risk stratification for postoperative AKI among the 40 million patients undergoing noncardiac surgery in the United States annually.^5^ To our knowledge, no consensus risk stratification algorithms or tools exist either before or after surgery. Improving risk stratification may be helpful for preoperative and perioperative management in the setting of noncardiac surgery.

Existing models to predict AKI provide moderate^6^ levels of accuracy,^7,8,9,10^ although they have not used consistent definitions of the AKI outcome, have used a mix of statistical and machine learning approaches, and have not uniformly focused on noncardiac surgery. For example, large studies of AKI after general or other noncardiac surgery demonstrated moderate predictive accuracy (eg, area under the receiver operating characteristic curve [AUC], 0.73-0.80), but predated current consensus standards on AKI definition.^11,12^ The lack of common definitions and methods underscores the need to compare performance across these various approaches. Furthermore, while some studies have used data from the electronic health record (EHR), they have not incorporated detailed physiological and clinical data (eg, vital signs, dosages of vasopressor medications, blood loss) collected intraoperatively. Because adding such data improves risk stratification for other postoperative complications,^13^ these data may also yield improvements in risk stratification for AKI.

In this study, we examined whether adding intraoperative data was associated with improved prediction of noncardiac postoperative AKI compared with models using administrative and preoperative clinical information alone. Furthermore, we compared performance across multiple statistical and machine learning approaches and definitions of AKI.

## Methods

### Study Data

Electronic health record data were collected on adult patients undergoing noncardiac surgery during an inpatient admission between January 1, 2014, and April 30, 2018, at the University of Pennsylvania Health System. We used code developed by the Multicenter Perioperative Outcomes Group that was run on University of Pennsylvania Health System Epic Clarity databases to standardize intraoperative and postoperative data and combined the data with administrative and preoperative data.^14^ Cohort data were randomly split by patient into derivation (60%), validation (20%), and test (20%) sets.^15^

The University of Pennsylvania Institutional Review Board approved the study design and granted a waiver of informed consent from study participants for secondary use of electronic health records. This study follows the Transparent Reporting of a Multivariable Prediction Model for Individual Prognosis or Diagnosis (TRIPOD) reporting guideline.^16^

### Study Population

Patients 18 years or older across 4 academic medical centers in University of Pennsylvania Health System during the study period were included if they underwent major noncardiac surgery. We identified noncardiac surgery using primary *Current Procedural Terminology* codes (10021-32999, 34001-69990)^17^ and restricted to major therapeutic procedures using Agency for Healthcare Research Quality Healthcare Cost Utilization Project Surgery Flag Software.^18^ We focused on noncardiac surgery because the association between preoperative and intraoperative variables and AKI likely differ for cardiac surgery owing to the use of cardiopulmonary bypass.

Patients who underwent multiple major surgical procedures during the same visit were excluded (4249 [5.4%] of surgical cases) to avoid overlap between preoperative and postoperative periods. In addition, patients were excluded if they did not have at least 1 preoperative and postoperative serum creatinine measurement (27 704 [35.5%] of surgical cases), had end-stage renal disease and underwent dialysis within the past year, had an elevated baseline serum creatinine level greater than or equal to 4.5 mg/dL (to convert to micromoles per liter, multiply by 88.4),^9^ or if they met criteria for AKI within the 7 days before surgery (additional details and billing codes in eMethods in the Supplement).

### Outcomes

Our primary outcome was the incidence of AKI within 7 days after surgery. For our primary analyses, we used the Kidney Disease Improving Global Outcomes guidelines for stage 1 AKI, defined as a serum creatinine level increase of 1.5 times baseline or of 0.3 mg/dL in a 48-hour period.^19^ We excluded the urine output criteria owing to concerns for poor specificity for AKI classification^20^ and the lack of reliable data in our data set. If discharge occurred earlier than 7 days after surgery and there was no evidence of AKI to date, an outcome of no AKI was assigned. Secondary outcomes included use of inpatient dialysis, a postsurgical length of stay of 7 or more days (to reflect a prolonged postsurgical stay), and in-hospital mortality (eMethods in the Supplement).

### Baseline Kidney Function Assessment

Baseline values were defined first as the lowest serum creatinine measurement value and estimated glomerular filtration rate value within 7 days before the start of surgery^21^ or, if no values were present, the most recent value up to 365 days before the surgery.^22^

### Variables

The unit of observation was an inpatient hospitalization for noncardiac surgery. Variables were split into 3 groups reflecting increasing inclusiveness of data: prehospitalization, preoperative, and perioperative variables. Prehospitalization variables included age, sex, race, and insurance type. Historical comorbidities were also included, derived from *International Classification of Diseases, Clinical Modification, Ninth Revision*, and *International Statistical Classification of Diseases, Clinical Modification, 10th Revision*, diagnostic codes.^23^ Preoperative variables combined the prehospitalization variables with clinical information related to the patient’s admission but before surgery, such as laboratory measurements, American Society of Anesthesiologists physical status,^24^ and surgical procedure type. To categorize operations, we used Agency for Healthcare Research Quality Healthcare Cost Utilization Project Clinical Classification Software to map each primary *Current Procedural Terminology* code to 244 unique procedure groups.^25^ Data for these variables were collected from the start of the admission up until the start of the surgical procedure. Perioperative variables added intraoperative data to preoperative variables. Intraoperative data included variables such as heart rate and blood pressure; fluid status, such as total fluid administration and estimated blood loss; and drug use, such as vasopressors and intraoperative rescue medications (eg, calcium chloride). Data for this category were collected between the start and end of the surgical procedure using timestamps in the EHR (full list of variables reported in the eAppendix in the Supplement).

### Missing Data on Variables

Because some variables contain data artifacts and extreme values, we set variables with values below the first percentile to the first percentile value and values greater than the 99th percentile to the 99th percentile value. After data cleaning, rates of missing data within observations ranged from 0.10% (ie, intraoperative heart rate) to 98.6% (ie, N-terminal pro b-type natriuretic peptide laboratory measurement) (eTable 1 in the Supplement). To avoid excluding observations that were missing data on predictor variables, we added dichotomous variables for each covariate that indicated whether an observation had a missing value. For observations with a missing indicator equal to 1, the missing covariate data were replaced with a fixed value.^26^ This approach allowed us to use a larger study sample while preserving information about present vs missing values. This approach is more flexible than general mean imputation and less stringent than the common missing-at-random assumption required in multiple imputation.

### Statistical Analysis

To examine improvements in predictive accuracy and risk stratification when adding more variables throughout the surgery encounter, we implemented models for each variable group (prehospitalization, preoperative, and perioperative) separately. We used 3 modeling approaches: logistic regression with elastic net selection, random forest, and gradient boosting machines (GBMs), which we applied to each definition of AKI. For random forest and GBM models, we used a randomized grid search using 3-folds across 30 iterations on our derivation data set for selecting optimal model parameters. For GBMs, we used decision trees as the weak learner with logistic regression for the loss function. Validation sets were used to evaluate, verify, and finalize our model parameters. Final model results are reported for the test sets of data only.

#### Model Performance

We compared differences between the development, validation, and test data sets and reported results of model performance using the test data sets (20% of sample). Categorical variables were compared using χ^2^ tests and continuous variables were compared using Mann-Whitney tests. Model performance was assessed using the AUC,^27^ which we calculated by comparing the AKI estimated from the models with observed AKI. We calculated 95% CIs using the method of DeLong et al^28^ with 1000 bootstrapping samples to test for significance between models. We compared model performance within each of the 3 modeling approaches for each of the 3 groups of variables (reflecting the progressive addition of data), as well as across the 3 modeling approaches when using the same group of data elements.

#### Risk Stratification

To illustrate implications for clinical utility, we stratified patients as high and low risk for Kidney Disease Improving Global Outcomes AKI and compared incidence rates of our primary and secondary outcomes associated with AKI. Patients were stratified into a high-risk category if their predicted risk for AKI was in the top 20% of the test data set population (n = 8494),^29^ with the remaining 80% of patients stratified into a low-risk category. Risk stratification was conducted on prehospitalization, preoperative, and perioperative data sets, examined for primary and secondary outcomes, and examined by patient encounters with and without events.

#### Sensitivity Analyses

We tested the sensitivity of our results to several data and modeling decisions, including using a super learner algorithm, classifying outlier data values as missing, by surgical type (eg, orthopedic, general, and neurologic), and alternative definitions of AKI (eMethods in the Supplement).^30,31,32^ Given the lack of an evidence-based definition of a high-risk probability value for AKI, the top 20% was arbitrarily selected and so we examined sensitivity to cutoff by using top 10% and top 30%.

Logistic regression with elastic net selection (PROC GLMSELECT) was implemented using SAS software, version 9.4 (SAS Institute Inc). Super Learner was implemented using the R, version 3.4.3 SuperLearner Package (R Foundation). All other code and predictive models (RandomForestClassifier, GradientBoostingClassifier) were conducted in Python, version 3.6 (Python Software Foundation), with Pandas 0.23.3 and Scikit-learn 0.19.1 libraries. Two-tailed tests were considered statistically significant at *P* < .05.

## Results

### Study Population

Of the 77 975 patients who underwent major noncardiac surgery, we identified 42 615 noncardiac surgical patient encounters that met study criteria (Table 1). Mean (SD) patient age was 57.9 (15.7) years, 23 943 (56.2%) patients were women, 27 857 (65.4%) patients were white, and 19 470 patients (45.7%) had commercial insurance. The most common surgery types were orthopedic (15 718 [36.9%]), general (8808 [20.7%]), and neurologic (6564 [15.4%]). Most patients were classified as American Society of Anesthesiologists physical status 3 (severe systemic disease) or 2 (mild systemic disease) before surgery.^24^ A total of 3859 patients (9.1%) had multiple operations during the study period. Of the study sample, 4318 patients (10.1%) experienced AKI (Table 2), which was similar across definitions (eTable 2 in the Supplement). In addition, 103 patients (0.2%) underwent inpatient dialysis, 8335 patients (19.6%) experienced a postoperative length of stay of 7 or more days, and 255 patients (0.6%) died in the hospital. Patient characteristics, rates of AKI, and other clinical outcomes did not exhibit substantial differences between derivation, validation, and test sets (Table 2).

**Table 1.** Patient Characteristics in the Model Derivation, Validation, and Test Sets^a^

Characteristic	No. (%)			
	All Visits (N = 42 615)	Set		
		Derivation (n = 25 616)	Validation (n = 8505)	Test (n = 8494)
Age, mean (SD), y	57.9 (15.7)	57.9 (15.6)	57.8 (15.9)	58 (15.6)
Women	23 943 (56.2)	14 438 (56.4)	4783 (56.2)	4722 (55.6)
Marital status				
Married	22 519 (52.8)	13 499 (52.7)	4500 (52.9)	4520 (53.2)
Single	12 707 (29.8)	7630 (29.8)	2564 (30.2)	2513 (29.6)
Other/unknown	7389 (17.3)	4487 (17.5)	1441 (16.9)	1461 (17.2)
Race				
White	27 857 (65.4)	16 717 (65.3)	5554 (65.3)	5586 (65.8)
Black	11 395 (26.7)	6874 (26.8)	2296 (27.0)	2225 (26.2)
Asian	934 (2.2)	545 (2.1)	186 (2.2)	203 (2.4)
Other/unknown	1034 (2.4)	626 (2.4)	205 (2.4)	203 (2.4)
Insurance				
Commercial	19 470 (45.7)	11 673 (45.6)	3857 (45.4)	3940 (46.4)
Medicare	16 978 (39.8)	10 233 (40.0)	3363 (39.5)	3382 (39.8)
Medicaid	5504 (12.9)	3336 (13.0)	1114 (13.1)	1054 (12.4)
Other	663 (1.6)	374 (1.5)	171 (2.0)	118 (1.4)
Surgery type				
Breast/dermatologic	2419 (5.7)	1426 (5.6)	498 (5.9)	495 (5.8)
Endocrine	482 (1.1)	292 (1.1)	93 (1.1)	97 (1.1)
General	8808 (20.7)	5259 (20.5)	1791 (21.1)	1758 (20.7)
Gynecologic	2344 (5.5)	1427 (5.6)	441 (5.2)	476 (5.6)
Neurologic	6564 (15.4)	3899 (15.2)	1398 (16.4)	1267 (14.9)
Obstetric	371 (0.9)	216 (0.8)	73 (0.9)	82 (1.0)
Orthopedic	15 718 (36.9)	9526 (37.2)	3082 (36.2)	3110 (36.6)
Thoracic	1495 (3.5)	914 (3.6)	275 (3.2)	306 (3.6)
Transplant	386 (0.9)	226 (0.9)	90 (1.1)	70 (0.8)
Urologic	1210 (2.8)	715 (2.8)	243 (2.9)	252 (3.0)
Vascular	1929 (4.5)	1161 (4.5)	357 (4.2)	411 (4.8)
Other	889 (2.1)	555 (2.2)	164 (1.9)	170 (2.0)
ASA physical status				
1	1349 (3.2)	770 (3.0)	283 (3.3)	296 (3.5)
2	18 515 (43.5)	11 106 (43.4)	3732 (43.9)	3677 (43.3)
3	21 068 (49.4)	12 710 (49.6)	4163 (49.0)	4195 (49.4)
≥4	1604 (3.8)	980 (3.8)	314 (3.7)	310 (3.7)
Unknown	79 (0.2)	50 (0.2)	13 (0.2)	16 (0.2)
Time to surgery, median (IQR), min	250 (170-835)	249 (169-806)	255 (173-840)	246 (170-913)
Surgery duration, median (IQR), min	121 (78-195)	121 (78-194)	119 (77-193)	123 (78-197)

Abbreviations: ASA, American Society of Anesthesiologists; IQR, interquartile range.

^a^ Baseline characteristics of the 42 615 patients who underwent major noncardiac surgery.

**Table 2.** Clinical Outcomes in the Model Derivation, Validation, and Test Sets^a^

Clinical Outcome	No. (%)			
	All Visits (N = 42 615)	Set		
		Derivation (n = 25 616)	Validation (n = 8505)	Test (n = 8494)
Acute kidney injury	4318 (10.1)	2655 (10.4)	818 (9.6)	845 (9.9)
Inpatient dialysis	103 (0.2)	54 (0.2)	17 (0.2)	32 (0.4)
Length of stay ≥7 d	8335 (19.6)	5032 (19.6)	1634 (19.2)	1669 (19.7)
In-hospital death	255 (0.6)	157 (0.6)	40 (0.5)	58 (0.7)

^a^ Primary and secondary clinical outcomes of the 42 615 patients who underwent major noncardiac surgery.

### Model Performance

Among the 8494 patients in the test set, 845 patients (9.9%) experienced Kidney Disease Improving Global Outcomes AKI (Table 2). Use of logistic regression with elastic net selection resulted in increasing AUCs as clinical variables were added (Figure): the AUC was 0.700 (95% CI, 0.681-0.719) with prehospitalization variables, 0.782 (95% CI, 0.765-0.799) with preoperative variables that included prehospitalization variables (*P* < .001 for AUC comparison vs model using prehospitalization variables only), and 0.790 (95% CI, 0.773-0.807) with perioperative variables that included intraoperative variables (*P* = .02 for AUC comparison vs model using preoperative variables only). The random forest models resulted in an AUC of 0.710 (95% CI, 0.690-0.728) with prehospitalization variables, a higher AUC of 0.787 (95% CI, 0.770-0.803) with preoperative variables (*P* < .001 for AUC comparison vs model using prehospitalization variables only), and the highest AUC of 0.808 (95% CI, 0.790-0.823) using perioperative variables (*P* < .001 for AUC comparison vs model using preoperative variables only). The GBM models generated the highest AUCs across all models with an AUC of 0.712 (95% CI, 0.694-0.731) using the prehospitalization variables, a higher AUC of 0.804 (95% CI, 0.788-0.819) with preoperative variables (*P* < .001 for AUC comparison vs model using prehospitalization variables only), and the highest AUC of 0.817 (95% CI, 0.802-0.832) when using perioperative variables (*P* < .001 for AUC comparison vs model using prehospitalization variables only). Full model performance across data sets, calibration curves, and variable coefficients and importance can be found in eTables 3-7 and the eFigure in the Supplement.

**Figure.**
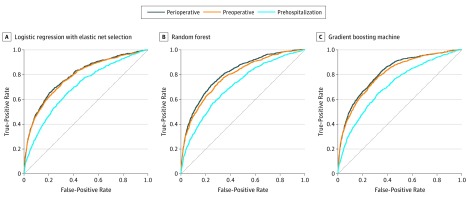
Comparison of the Performance of 3 Modeling Approaches Using Prehospitalization, Preoperative, and Perioperative Data for Acute Kidney Injury Logistic regression with elastic net selection (A), random forest (B), and gradient boosting machine (C) methods used for modeling. The cyan line is the model containing prehospitalization variables. The orange line is the model using preoperative variables (including prehospitalization variables). The navy line is the model using perioperative data (including preoperative and prehospitalization variables). Receiver operating characteristic curves (AUCs) for each model using prehospitalization, preoperative, and perioperative variable groups are shown in the test set. The AUC or C-statistic is calculated along with 95% CIs. The DeLong et al^28^ test indicates a significant difference between model AUCs (*P* < .001).

### Risk Stratification

A total of 1699 of the 8494 patients (20.0%) were classified as high risk and 6795 patients (80.0%) were classified as low risk, using the GBM model (Table 3 and Table 4). We applied this risk stratification to each group of variables separately (reflecting progressive addition of clinical variables) and compared classification. Although the improvement in discrimination was statistically significant when adding perioperative data, the improvement did not appear to be clinically significant. In particular, the AKI rate among patients classified as high risk improved from 29.1% to 30.0%; however, only a net of 15 patients were appropriately reclassified as high risk (ie, 67 patients were reclassified appropriately as high risk, but 52 patients were reclassified inappropriately as low risk) and an additional net of 15 patients were appropriately reclassified as low risk (ie, 329 patients were appropriately reclassified as low risk but 314 patients were inappropriately reclassified as high risk) (Table 3).

**Table 3.** Acute Kidney Injury Risk as Predicted by Models That Add and Do Not Add Intraoperative Data in Test Data Set^a^

GBM Preoperative Model	No. (%)		
	GBM-Perioperative Model^a^		Total, No.
	Low Risk	High Risk	
**Low Risk**^**b**^			
Encounters	6414 (94.4)	381 (5.6)	6795
Events	283 (80.9)	67 (19.1)	350
Nonevents	6131 (95.1)	314 (4.9)	6445
Proportion of encounters with events	0.044	0.176	0.052
**High Risk**^**b**^			
Encounters	381 (22.4)	1318 (77.6)	1699
Events	52 (10.5)	443 (89.5)	495
Nonevents	329 (27.3)	875 (72.7)	1204
Proportion of encounters with events	0.136	0.336	0.291

Abbreviation: GBM, gradient boosting machine.

^a^ Risk stratification of GBM models in the test set for the outcome of acute kidney injury using preoperative and perioperative data in the test data set (n = 8494). For the GBM model using the perioperative model, the overall proportion of encounters with events was 0.300 and 0.049 for high- and low-risk groups, respectively.

^b^ High risk was defined as the top 20% of predicted risk. Low risk was defined as the bottom 80% of predicted risk.

**Table 4.** Acute Kidney Injury Risk Stratification in Test Data Set and Rates of Clinical Outcomes by Variable Group^a^

GBM Acute Kidney Injury Model Risk Stratification^b^	Sample (n = 8494)	No. (%)			
		Acute Kidney Injury (n = 845)	Inpatient Dialysis (n = 32)	Postoperative Length of Stay ≥7 d (n = 1669)	In-Hospital Death (n = 58)
**Prehospitalization Variables**					
High risk	1699	378 (22.3)	22 (1.3)	567 (33.4)	34 (2.0)
Low risk	6795	467 (6.9)	10 (0.2)	1102 (16.2)	24 (0.4)
**Preoperative Variables**					
High risk	1699	495 (29.1)	28 (1.7)	738 (43.4)	40 (2.4)
Low risk	6795	350 (5.2)	4 (0.1)	931 (13.7)	18 (0.3)
**Perioperative Variables**					
High risk	1699	510 (30.0)	30 (1.8)	774 (45.6)	51 (3.0)
Low risk	6795	335 (4.9)	2 (0.03)	895 (13.2)	7 (0.1)

Abbreviation: GBM, gradient-boosting machine.

^a^ Risk stratification of GBM models in the test data set (n = 8494). Incidence rates of primary and secondary clinical outcomes were calculated from sample totals. In-patient dialysis was defined using *International Classification of Diseases, Ninth Revision*,* Clinical Modification* procedure codes (eMethods in the Supplement).

^b^ High risk was defined as the top 20% of predicted risk. Low risk was defined as the bottom 80% of predicted risk.

The small improvements were concordant across primary and secondary outcomes (Table 4). Rates of Kidney Disease Improving Global Outcomes AKI in the high-risk groups increased as more data were added (prehospitalization, 22.3%; preoperative, 29.1%; perioperative, 30.0%). Rates of secondary outcomes increased similarly: inpatient dialysis (prehospitalization, 1.3%; preoperative, 1.7%; perioperative, 1.8%), postoperative length of stay greater than or equal to 7 days (prehospitalization, 33.4%; preoperative, 43.4%; perioperative, 45.6%), and in-hospital death (prehospitalization, 2.0%; preoperative, 2.4%; perioperative, 3.0%). The largest increases were observed after adding preoperative data, while smaller increases were observed after adding intraoperative data.

### Sensitivity Analyses

The results of several sensitivity analyses were consistent with our main results (eTables 8-12 in the Supplement).

## Discussion

The findings of this study suggest that clinical EHR data can be used to develop reasonably accurate predictive models for risk-stratifying adults undergoing major noncardiac surgery for postoperative AKI. Model performance increased as more clinical information was incorporated, with the largest performance gains noted when preoperative data were added. This finding was robust to different modeling techniques and definitions of AKI.

However, the gains in accuracy from adding intraoperative data to preoperative data were modest at best, showing only marginal gains in the AUC, and did not seem to be clinically meaningful. These results were similarly reflected in risk stratification. For example, of the entire test set population of 8494 patients, only 30 were appropriately reclassified as high or low risk when adding perioperative data. This finding may suggest that adding intraoperative data to risk stratification models for AKI may not yield substantial benefits relative to the complexity in implementation. This is further highlighted by the contrast in results for models of other postoperative complications, such as in-hospital mortality, for which the addition of intraoperative data yields substantial improvements in risk stratification.^13^

Although our models did not demonstrate substantially higher discrimination on average across the entire study population, there may be subgroups of patients for whom addition of intraoperative data improves risk stratification in a clinically meaningful fashion. Additional research exploring subgroups is underway as part of a broader effort to implement such algorithms into practice. One feature of the models we used is that they are suited to implementation in electronic systems that receive or pull data from the EHR.

Another contribution of this study was to implement multiple statistical and machine learning methods as well as use of multiple definitions of AKI as the primary outcome. This approach suggests that our results may reflect the accuracy of risk stratification models for AKI and highlights that variability in modeling approach and AKI outcome definitions may be unlikely to explain differences in discrimination (ie, AUCs ranging from 0.73 to 0.80) in previous studies.^8,9,10^

### Limitations

The study has several limitations. First, this was a single-institution study and the availability of EHR data as well as practice patterns may vary at other institutions. However, we used data from multiple hospitals within a health system with different surgery and anesthesia groups and clinicians. Furthermore, the intraoperative data that we used are likely captured as part of routine monitoring of patients while in surgery. Third, our follow-up period was limited to the hospital setting and there may have been limited documentation of other important clinical outcomes. We did not capture longitudinal outcomes, which may affect the ability to risk stratify for other important, longer-term outcomes. Fourth, we did not have reliable data on urine output, which could have led to incomplete identification of AKI.

## Conclusions

The findings of this study suggest that EHR data can be used to accurately stratify patients at risk of perioperative AKI. However, the modest improvements in performance from adding intraoperative data should be weighed against clinical utility and examination of whether particular subgroups may benefit from the addition requires further research.

## Notes

Supplement. **eMethods.** Detailed Methods **eAppendix.** Variables **eTable 1.** Rates of Missing Data in Variables **eTable 2.** Extended Clinical Outcomes in the Model Derivation, Validation, and Test Sets **eTable 3.** Full Model Performance in the Model Derivation, Validation, and Test Sets **eFigure.** Model Calibration Curves **eTable 4.** Logistic Regression With Elastic Net Selection Estimates Using Prehospitalization Data **eTable 5.** Logistic Regression With Elastic Net Selection Estimates Using Preoperative Data **eTable 6.** Logistic Regression With Elastic Net Selection Estimates Using Perioperative Data **eTable 7.** Feature Importance for Random Forest and Gradient Boosting Machine Models **eTable 8.** Super Learner Model Performance for Acute Kidney Injury **eTable 9.** Model Performance for Acute Kidney Injury When Setting Extreme Covariate Values to Missing **eTable 10.** Model Performance in Top 3 Surgical Subgroups in Test Dataset **eTable 11.** Model Results by Acute Kidney Injury Definition and Modeling Approach **eTable 12.** Acute Kidney Injury Risk Stratification Using Alternate Definitions of High Risk in Test Dataset – Rates of Clinical Outcomes by Variable Group **eReferences.**
