# Supplementary material for: Risk Stratification for Postoperative Acute Kidney Injury in Major Noncardiac Surgery Using Preoperative and Intraoperative Data
Source: JAMA Netw Open. 2019 Dec 6;2(12):e1916921. doi: 10.1001/jamanetworkopen.2019.16921 (PMC6902769; doi:10.1001/jamanetworkopen.2019.16921)
Supplement: Supplement. — eMethods. Detailed Methods eAppendix. Variables eTable 1. Rates of Missing Data in Variables eTable 2. Extended Clinical Outcomes in the Model Derivation, Validation, and Test Sets eTable 3. Full Model Performance in the Model Derivation, Validation, and Test Sets eFigure. Model Calibration Curves eTable 4. Logistic Regression With Elastic Net Selection Estimates Using Prehospitalization Data eTable 5. Logistic Regression With Elastic Net Selection Estimates Using Preoperative Data eTable 6. Logistic Regression With Elastic Net Selection Estimates Using Perioperative Data eTable 7. Feature Importance for Random Forest and Gradient Boosting Machine Models eTable 8. Super Learner Model Performance for Acute Kidney Injury eTable 9. Model Performance for Acute Kidney Injury When Setting Extreme Covariate Values to Missing eTable 10. Model Performance in Top 3 Surgical Subgroups in Test Dataset eTable 11. Model Results by Acute Kidney Injury Definition and Modeling Approach eTable 12. Acute Kidney Injury Risk Stratification Using Alternate Definitions of High Risk in Test Dataset – Rates of Clinical Outcomes by Variable Group eReferences. [file jamanetwopen-2-e1916921-s001.pdf]

Supplementary Online Content

Lei VJ, Luong T, Shan E, et al. Risk stratification for postoperative acute kidney injury in major noncardiac surgery using preoperative and intraoperative data. *JAMA Netw Open*. 2019;2(12):e1916921. doi:10.1001/jamanetworkopen.2019.16921

eMethods. Detailed Methods

eAppendix. Variables

eTable 1. Rates of Missing Data in Variables

eTable 2. Extended Clinical Outcomes in the Model Derivation, Validation, and Test Sets

eTable 3. Full Model Performance in the Model Derivation, Validation, and Test Sets

eFigure. Model Calibration Curves

eTable 4. Logistic Regression With Elastic Net Selection Estimates Using Prehospitalization Data

eTable 5. Logistic Regression With Elastic Net Selection Estimates Using Preoperative Data

eTable 6. Logistic Regression With Elastic Net Selection Estimates Using Perioperative Data

eTable 7. Feature Importance for Random Forest and Gradient Boosting Machine Models

eTable 8. Super Learner Model Performance for Acute Kidney Injury

eTable 9. Model Performance for Acute Kidney Injury When Setting Extreme Covariate Values to Missing

eTable 10. Model Performance in Top 3 Surgical Subgroups in Test Dataset

eTable 11. Model Results by Acute Kidney Injury Definition and Modeling Approach

eTable 12. Acute Kidney Injury Risk Stratification Using Alternate Definitions of High Risk in Test Dataset – Rates of Clinical Outcomes by Variable Group

eReferences

This supplementary material has been provided by the authors to give readers additional information about their work.

© 2019 Lei VJ et al. *JAMA Network Open.*

eMethods. Detailed Methods

Study Sample

Inclusion Criteria: Individuals (1) age 18 or greater; (2) recorded inpatient hospital admission; (3) undergoing a major noncardiac surgery

Exclusion Criteria: Individuals with (1) multiple major surgeries during hospitalization; (2) did not have at least one preoperative and postoperative serum creatinine (SCr) measurement; (3) end stage renal disease (ESRD) undergoing dialysis within the past year*; (4) high baseline SCr of 4.5 mg/dl or greater (because clinical criteria for AKI based on SCr elevation may not apply to these patients); or (5) experienced acute kidney injury up to seven days before surgery

CONSORT Diagram

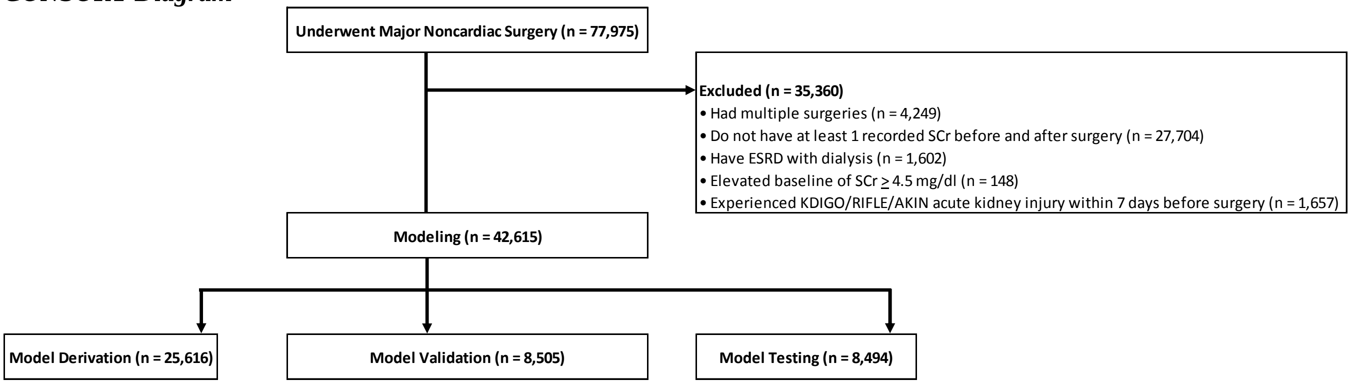

Abbreviations: SCr, serum creatinine; ESRD, End stage renal disease; KDIGO, Kidney Disease: Improve Global Outcomes; RIFLE, Risk, Injury, Failure, Loss of kidney function, End stage kidney disease; AKIN, Acute Kidney Injury Network

****Exclusion criteria: Billing Codes for ESRD undergoing Dialysis[1]***

Patients were excluded from the study if they previously had a diagnosis or billing code within 1 year of index hospitalization.

ICD-9-CM Diagnosis Codes

585.6 End stage renal disease
996.81 Complications of transplanted kidney
V42.0 Kidney replaced by transplant
V45.1 Postsurgical renal dialysis status
V56.0 Encounter for extracorporeal dialysis
V56.1 Fitting and adjustment of extracorporeal dialysis catheter
V56.2 Fitting and adjustment of peritoneal dialysis catheter
V56.3 Encounter for adequacy testing for dialysis
V56.31 Encounter for adequacy testing for hemodialysis
V56.32 Encounter for adequacy testing for peritoneal dialysis
V56.8 Encounter for other dialysis
E879.1 Kidney dialysis as the cause of abnormal reaction of patient, or of later complication, without mention of misadventure at time of procedure

ICD-10-CM Diagnosis Codes

T86.10 Unspecified complication of kidney transplant
T86.11 Kidney transplant rejection
T86.12 Kidney transplant failure
T86.13 Kidney transplant infection
T86.19 Other complication of kidney transplant
Z99.2 Dependence on renal dialysis

CPT Procedure Codes

90935 Hemodialysis one evaluation
90937 Hemodialysis repeated eval
90940 Hemodialysis access study
90945 Dialysis one evaluation

© 2019 Lei VJ et al. *JAMA Network Open.*

90947 Dialysis repeated eval
90951 ESRD serv 4 visits p mo <2yr
90952 ESRD serv 2-3 vsts p mo <2yr
90953 ESRD serv 1 visit p mo <2yrs
90954 ESRD serv 4 vsts p mo 2-11
90955 ESRD srv 2-3 vsts p mo 2-11
90956 ESRD srv 1 visit p mo 2-11
90957 ESRD srv 4 vsts p mo 12-19
90958 ESRD srv 2-3 vsts p mo 12-19
90959 ESRD serv 1 vst p mo 12-19
90960 ESRD srv 4 visits p mo 20+
90961 ESRD srv 2-3 vsts p mo 20+
90962 ESRD serv 1 visit p mo 20+
90963 ESRD home pt serv p mo <2yrs
90964 ESRD home pt serv p mo 2-11
90965 ESRD home pt serv p mo 12-19
90966 ESRD home pt serv p mo 20+
90967 ESRD home pt serv p day <2
90968 ESRD home pt srv p day 2-11
90969 ESRD home pt srv p day 12-19
90970 ESRD home pt serv p day 20+
90989 Dialysis training complete
90993 Dialysis training incompl
90997 Hemoperfusion
90999 Dialysis procedure

Secondary Outcomes
Secondary outcomes included inpatient dialysis, a post-surgical length of stay ≥ 7 days (to reflect a prolonged post-surgical stay), and all-cause in-hospital death. Inpatient dialysis was identified if a procedure code in the list below was present during index hospitalization. The post-surgical length of stay threshold of ≥ 7 days was selected as a marker for prolonged length of stay. In the absence of a well-defined threshold in the literature, we selected 7 days after surgery because it aligned with our post-operative AKI definition (which was defined up to 7 days after surgery). All-cause in-hospital death was defined as patient death anytime between the end of surgery and before discharge of index hospitalization.

Inpatient Dialysis Procedure Codes
ICD-9-PCS Procedure Codes
39.95 Hemodialysis

ICD-10-PCS Procedure Codes
5A1D60Z Performance of Urinary Filtration, Multiple
5A1D70Z Performance of Urinary Filtration, Intermittent, Less than 6 Hours Per Day
5A1D00Z Performance of Urinary Filtration, Single
5A1D90Z Performance of Urinary Filtration, Continuous, Greater than 18 hours Per Day
5A1D80Z Performance of Urinary Filtration, Prolonged Intermittent, 6-18 hours Per Day

© 2019 Lei VJ et al. *JAMA Network Open.*

Sensitivity Analyses
We tested the sensitivity of our results to several data and modeling decisions.

1) Ensembling Models - Super Learner
In response to an editor's comment, we examined the performance difference between our models and an ensemble technique like Super Learner,[2] which are beginning to become more common. Algorithms chosen for analysis were penalized logistic regression (glmnet), gradient boosting machine (gbm), XGboost (xgboost), and random forest (randomForest).

2) Alternate Method Handling for Extreme and Artifact Values
In response to an editor's comment, we test whether results were sensitive to treating outlier and extreme variable data as missing instead of our main approach. Values below the 1st percentile and values greater than the 99th percentile were set to missing for this analysis. All modeling and analysis were consistent otherwise.

3) Surgical Subgroup Analysis
In response to a reviewer comment, we examine if model performance differed by surgical specialty-specific models. Models were trained and tested in each respective subgroups. Results were compared to the main analysis.

4) Alternate Acute Kidney Injury Definitions
To address multiple definitions of AKI by professional societies, we also used two other definitions: (1) Risk, Injury, and Failure; and Loss; and End-stage kidney disease (RIFLE)[3] classification of risk--developed by Acute Dialysis Quality Initiative--was defined as an increase of SCr by 1.5 times, or estimated glomerular filtration rate (eGFR) decrease by 25%, and (2) Acute Kidney Injury Network (AKIN) stage 1 was defined as an absolute increase in serum creatinine by 0.3 mg/dl (26.4 μmol/l) or a 50% increase in serum creatinine (i.e. 1.5-fold from baseline).[4]
eGFR was calculated using the Chronic Kidney Disease Epidemiology Collaboration (CKD-EPI) equation.[5] Models for main analysis will be reproduced with the two alternate definitions of AKI.

5) High Risk Stratification Cutoff Analysis
Given the lack of an evidence-based definition of a high-risk probability value for AKI, the top 20% was arbitrarily selected and we examined sensitivity to cutoff by used top 10% and top 30%.

© 2019 Lei VJ et al. *JAMA Network Open.*

eAppendix. Variables

A total of 339 baseline, preoperative, and intraoperative variables were constructed. Final models in main and sensitivity analyses were derived using a set of components of these variables. All categorical variables were one-hot encoded to unique binary variables.

Baseline Variables (35)
Age, sex, marital status, race, insurance, Elixhauser comorbidities (30)[6]

Preoperative Variables (274)
Last known laboratory measurement value up to 30 days (26; NT-proBNP, creatine kinase, GFR non-African American, glucose POC, glucose level, sodium level, potassium level, chloride level, creatinine level, blood urea nitrogen, bicarbonate venous, whole blood glucose, hemoglobin, hematocrit, white blood cell, red blood cell, platelet count, alanine aminotransferase, bilirubin total, aspartate aminotransferase, alkaline phosphatase, bilirubin direct, bilirubin indirect, international normalization ratio, prothrombin time, partial thromboplastin time); labs ordered (2; troponin-I point of care, troponin-T); ASA physical status; Agency for Healthcare Research and Quality Healthcare Cost and Utilization Project's Clinical Classification Software for Procedures (244)[7]; High Risk Surgery[8]

Intraoperative Variables (30)
Time to surgery (min); surgery duration (min); urine output (ml); total of crystalloid administration (ml); estimated blood loss (ml); arterial line placement; Minutes under mean arterial pressure of 50; Minutes under mean arterial pressure of 60; Intraoperative fluid administration (5; platelets, fresh frozen plasma, packed red blood cells, salvaged red blood cells, whole blood autologous); Total dose medication in milligrams (5; epinephrine, phenylephrine, norepinephrine, ephedrine, vasopressin); Intraoperative medication administration (3; sodium bicarbonate, atropine, calcium chloride); Lowest recorded vital sign value (4; heart rate, mean arterial pressure, systolic blood pressure, diastolic blood pressure); Highest recorded vital sign value (4; heart rate, mean arterial pressure, systolic blood pressure, diastolic blood pressure); Mean (calculated) vital sign value (4; heart rate, mean arterial pressure, systolic blood pressure, diastolic blood pressure); Delta (Highest – Lowest) vital sign value (3; heart rate, systolic blood pressure, diastolic blood pressure)

© 2019 Lei VJ et al. *JAMA Network Open.*

eTable 1. Rates of Missing Data in Variables

The number of observations with missing data for variables were calculated in the study sample (n = 42,615). The table below shows the number of observations with missing data only for the variables that contain missing data.

**Source**	**Variable**	**Data Type**	**Number of Observations with Missing Values**	**% Observations with Missing Values**
Preoperative	NT-proBNP	continuous	40,227	98.6
Preoperative	Creatine Kinase	continuous	39,900	97.8
Preoperative	GFR Non-African American	continuous	11,716	28.7
Preoperative	Glucose POC	continuous	39,650	97.2
Preoperative	Glucose Level	continuous	3,596	8.8
Preoperative	Sodium Level	continuous	4,109	10.1
Preoperative	Potassium Level	continuous	4,248	10.4
Preoperative	Chloride Level	continuous	4,265	10.5
Preoperative	Creatinine Level	continuous	4,063	10.0
Preoperative	Blood Urea Nitrogen	continuous	4,078	10.0
Preoperative	Bicarbonate Venous	continuous	37,846	92.8
Preoperative	Whole Blood Glucose	continuous	38,227	93.7
Preoperative	Hemoglobin	continuous	3,911	9.6
Preoperative	Hematocrit	continuous	3,909	9.6
Preoperative	White Blood Cell	continuous	4,271	10.5
Preoperative	Red Blood Cell	continuous	4,268	10.5
Preoperative	Platelet Count	continuous	3,988	9.8
Preoperative	Alanine Aminotransferase	continuous	18,206	44.6
Preoperative	Bilirubin Total	continuous	18,150	44.5
Preoperative	Aspartate Aminotransferase	continuous	18,216	44.7
Preoperative	Alkaline Phosphatase	continuous	18,171	44.6
Preoperative	Bilirubin Direct	continuous	34,135	83.7
Preoperative	Bilirubin Indirect	continuous	34,170	83.8
Preoperative	International Normalization Ratio	continuous	8,473	20.8
Preoperative	Prothrombin Time	continuous	8,475	20.8
Preoperative	Partial Thromboplastin Time	continuous	8,948	21.9
Intraoperative	Max estimated blood loss	continuous	9,410	23.1
Intraoperative	Max urine output	continuous	15,784	38.7
Intraoperative	Min heart rate	continuous	23	0.1
Intraoperative	Max heart rate	continuous	23	0.1
Intraoperative	Mean heart rate	continuous	23	0.1
Intraoperative	Delta heart rate	continuous	23	0.1
Intraoperative	Min systolic blood pressure	continuous	369	0.9
Intraoperative	Max systolic blood pressure	continuous	369	0.9
Intraoperative	Mean systolic blood pressure	continuous	369	0.9
Intraoperative	Delta systolic blood pressure	continuous	369	0.9
Intraoperative	Min diastolic blood pressure	continuous	376	0.9
Intraoperative	Max diastolic blood pressure	continuous	376	0.9
Intraoperative	Mean diastolic blood pressure	continuous	376	0.9
Intraoperative	Delta diastolic blood pressure	continuous	376	0.9
Intraoperative	Min mean arterial pressure	continuous	21	0.1
Intraoperative	Max mean arterial pressure	continuous	21	0.1
Intraoperative	Mean mean arterial pressure	continuous	21	0.1
Intraoperative	Minutes under mean arterial pressure of 50mmHg	continuous	21	0.1
Intraoperative	Minutes under mean arterial pressure of 60mmHg	continuous	21	0.1
Elixhauser Comorbidity	AIDS HIV	binary	244	0.6
Elixhauser Comorbidity	Alcohol abuse	binary	244	0.6
Elixhauser Comorbidity	Blood loss anemia	binary	244	0.6
Elixhauser Comorbidity	Cardiac arrhythmias	binary	244	0.6
Elixhauser Comorbidity	Chronic pulmonary disorders	binary	244	0.6

Elixhauser Comorbidity	Coagulopathy	binary	244	0.6
Elixhauser Comorbidity	Congestive heart failure	binary	244	0.6
Elixhauser Comorbidity	Deficiency anemia	binary	244	0.6
Elixhauser Comorbidity	Depression	binary	244	0.6
Elixhauser Comorbidity	Diabetes, complicated	binary	244	0.6
Elixhauser Comorbidity	Diabetes, uncomplicated	binary	244	0.6
Elixhauser Comorbidity	Drug abuse	binary	244	0.6
Elixhauser Comorbidity	Fluid and electrolyte disorders	binary	244	0.6
Elixhauser Comorbidity	Hypertension, complicated	binary	244	0.6
Elixhauser Comorbidity	Hypertension, uncomplicated	binary	244	0.6
Elixhauser Comorbidity	Hypothyroidism	binary	244	0.6
Elixhauser Comorbidity	Liver disease	binary	244	0.6
Elixhauser Comorbidity	Lymphoma	binary	244	0.6
Elixhauser Comorbidity	Metastatic cancer	binary	244	0.6
Elixhauser Comorbidity	Obesity	binary	244	0.6
Elixhauser Comorbidity	Other neurological disorders	binary	244	0.6
Elixhauser Comorbidity	Paralysis	binary	244	0.6
Elixhauser Comorbidity	Peptic ulcer disease	binary	244	0.6
Elixhauser Comorbidity	Peripheral vascular disorders	binary	244	0.6
Elixhauser Comorbidity	Psychoses	binary	244	0.6
Elixhauser Comorbidity	Pulmonary circulation	binary	244	0.6
Elixhauser Comorbidity	Rheumatoid arthritis/collagen vascular disease	binary	244	0.6
Elixhauser Comorbidity	Solid tumor without metastasis	binary	244	0.6
Elixhauser Comorbidity	Valvular disease	binary	244	0.6
Elixhauser Comorbidity	Weight loss	binary	244	0.6

© 2019 Lei VJ et al. *JAMA Network Open.*

eTable 2. Extended Clinical Outcomes in the Model Derivation, Validation, and Test Sets

Primary and secondary clinical outcomes of the 42,615 patients who underwent major noncardiac surgery.

		Set		
**Clinical Outcome**	**All Visits (n = 42,615)**	**Derivation (n = 25,616)**	**Validation (n = 8,505)**	**Test (n = 8,494)**
KDIGO Acute Kidney Injury, No. (%)	4,318 (10.1)	2,655 (10.4)	818 (9.6)	845 (10)
RIFLE Acute Kidney Injury, No. (%)	4,206 (9.9)	2,543 (9.9)	806 (9.5)	857 (10.1)
AKIN Acute Kidney Injury, No. (%)	4,177 (9.8)	2,574 (10.1)	784 (9.2)	819 (9.6)
Inpatient Dialysis, No. (%)	103 (0.2)	54 (0.2)	17 (0.2)	32 (0.4)
Prolonged Length of Stay, No. (%)	8,335 (19.6)	5,032 (19.6)	1,634 (19.2)	1,669 (19.7)
In-Hospital Death, No. (%)	255 (0.6)	157 (0.6)	40 (0.5)	58 (0.7)

Abbreviations: KDIGO, Kidney Disease: Improve Global Outcomes; RIFLE, Risk, Injury, Failure, Loss of kidney function, End stage kidney disease; AKIN, Acute Kidney Injury Network

© 2019 Lei VJ et al. *JAMA Network Open.*

eTable 3. Full Model Performance in the Model Derivation, Validation, and Test Sets

Models	Derivation Set (n = 25,616), AUC (95% CI)	Validation Set (n = 8,505), AUC (95% CI)	Test Set (n = 8,494), AUC (95% CI)
Logistic Regression with Elastic Net Selection			
Prehospitalization	0.695 (0.685, 0.706)	0.706 (0.687, 0.725)	0.700 (0.681, 0.719)
Preoperative	0.765 (0.756, 0.775)	0.779 (0.762, 0.796)	0.782 (0.765, 0.799)
Perioperative	0.777 (0.767, 0.786)	0.784 (0.767, 0.801)	0.790 (0.773, 0.807)
Random Forest			
Prehospitalization	0.740 (0.731, 0.750)	0.712 (0.693, 0.730)	0.710 (0.691, 0.728)
Preoperative	0.924 (0.920, 0.928)	0.789 (0.771, 0.807)	0.787 (0.769, 0.803)
Perioperative	0.951 (0.948, 0.954)	0.805 (0.789, 0.820)	0.808 (0.792, 0.824)
Gradient Boosting Machine			
Prehospitalization	0.725 (0.714, 0.735)	0.713 (0.694, 0.731)	0.712 (0.695, 0.731)
Preoperative	0.825 (0.817, 0.833)	0.803 (0.787, 0.819)	0.804 (0.788, 0.819)
Perioperative	0.848 (0.841, 0.855)	0.812 (0.797, 0.829)	0.817 (0.802, 0.832)

Abbreviations: AUC, Area under the receiver operating characteristics curve; CI, confidence interval

© 2019 Lei VJ et al. *JAMA Network Open.*

eFigure. Model Calibration Curves

Model calibration curves for each model is plotted below for the outcome acute kidney injury in test dataset (n = 8,494).

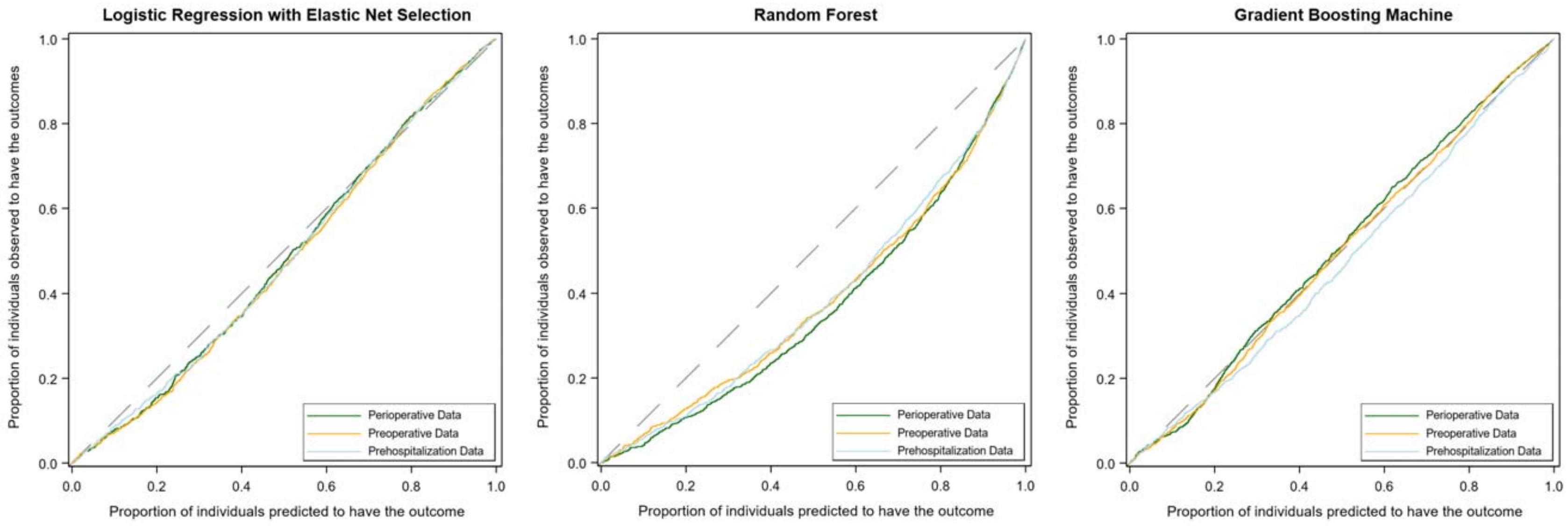

© 2019 Lei VJ et al. *JAMA Network Open.*

eTable 4. Logistic Regression with Elastic Net Selection Estimates using Prehospitalization Data

The final model for the logistic regression with elastic net selection using prehospitalization data (which includes prehospitalization and preoperative data) consisted of 42 variables.

Variable Group	Variable Name	Type	Reference	Odds Ratio (95% CI)	p-value
	Intercept		N/A	0.02(0.02, 0.03)	<.001
Prehospitalization	Age	Continuous	N/A	1.02(1.01, 1.02)	<.001
	Gender: MALE	Binary	FEMALE	1.36(1.24, 1.49)	<.001
	Marital Status: OTHER/UNKNOWN	Categorical	MARRIED	1.06(0.94, 1.19)	0.347
	Marital Status: SINGLE	Categorical	MARRIED	1.07(0.96, 1.2)	0.22
	Race: BLACK OR AFRICAN AMERICAN	Categorical	WHITE	1.23(1.1, 1.37)	<.001
	Race: ASIAN	Categorical	WHITE	0.87(0.62, 1.2)	0.39
	Race: OTHER/UNKNOWN	Categorical	WHITE	1.3(1.09, 1.55)	0.004
	Insurance: MEDICAID	Categorical	COMMERCIAL	1.21(1.04, 1.41)	0.013
	Insurance: MEDICARE	Categorical	COMMERCIAL	1.23(1.1, 1.38)	<.001
	Insurance: OTHER	Categorical	COMMERCIAL	4.43(3.38, 5.8)	<.001
	Elixhauser_AIDS_HIV	Binary	No	0.5(0.26, 0.97)	0.039
	Elixhauser_Alcohol_abuse	Binary	No	1.09(0.81, 1.46)	0.579
	Elixhauser_Blood_loss_anemia	Binary	No	0.99(0.7, 1.4)	0.963
	Elixhauser_Cardiac_arrhythmias	Binary	No	1.06(0.93, 1.21)	0.376
	Elixhauser_Chronic_pulmonary_disease	Binary	No	1.21(1.06, 1.38)	0.006
	Elixhauser_Coagulopathy	Binary	No	1.09(0.87, 1.37)	0.455
	Elixhauser_Congestive_heart_failure	Binary	No	1.58(1.31, 1.9)	<.001
	Elixhauser_Deficiency_anemia	Binary	No	1.14(0.9, 1.46)	0.282
	Elixhauser_Depression	Binary	No	0.82(0.7, 0.96)	0.016
	Elixhauser_Diabetes_complicated	Binary	No	1.01(0.84, 1.22)	0.924
	Elixhauser_Diabetes_uncomplicated	Binary	No	1.04(0.9, 1.2)	0.619
	Elixhauser_Drug_abuse	Binary	No	0.81(0.61, 1.07)	0.142
	Elixhauser_Fluid_and_electrolyte_disorders	Binary	No	1.36(1.18, 1.58)	<.001
	Elixhauser_Hypertension_complicated	Binary	No	0.91(0.73, 1.13)	0.399
	Elixhauser_Hypertension_uncomplicated	Binary	No	1.02(0.92, 1.13)	0.760
	Elixhauser_Hypothyroidism	Binary	No	0.94(0.8, 1.11)	0.465
	Elixhauser_Liver_disease	Binary	No	2.19(1.83, 2.63)	<.001
	Elixhauser_Lymphoma	Binary	No	1.19(0.84, 1.7)	0.321
	Elixhauser_Metastatic_cancer	Binary	No	0.87(0.71, 1.07)	0.191
	Elixhauser_Obesity	Binary	No	0.99(0.87, 1.12)	0.828
	Elixhauser_Other_neurological_disorders	Binary	No	0.75(0.6, 0.94)	0.011
	Elixhauser_Paralysis	Binary	No	0.93(0.59, 1.44)	0.735
	Elixhauser_Peptic_ulcer_disease	Binary	No	0.87(0.56, 1.36)	0.542
	Elixhauser_Peripheral_vascular_disease	Binary	No	1.01(0.87, 1.19)	0.856
	Elixhauser_Psychoses	Binary	No	1.01(0.58, 1.74)	0.982
	Elixhauser_Pulmonary_circulation	Binary	No	1.28(1.02, 1.59)	0.031
	Elixhauser_Renal_failure	Binary	No	2.59(2.14, 3.14)	<.001
	Elixhauser_Rheumatoid_arthritis_	Binary	No	0.93(0.74, 1.18)	0.552
	Elixhauser_Solid_tumor_without_metastasis	Binary	No	1.43(1.28, 1.6)	<.001
	Elixhauser_Valvular_disease	Binary	No	0.98(0.82, 1.17)	0.82
	Elixhauser_Weight_loss	Binary	No	1.21(1, 1.47)	0.049
	missing_Hxdiagnosis	Binary	No	1(0.54, 1.84)	0.995

© 2019 Lei VJ et al. *JAMA Network Open.*

eTable 5. Logistic Regression with Elastic Net Selection Estimates using Preoperative Data

The final model for the logistic regression with elastic net selection using preoperative data (which includes prehospitalization and preoperative data) consisted of 14 variables.

Variable Group	Variable Name	Type	Reference	Odds Ratio (95% CI)	p-value
	Intercept		N/A	0.21(0.11, 0.38)	<.001
Prehospitalization	Age	Continuous	N/A	1.01(1.01, 1.01)	<.001
	Elixahauser_Congestive_Heart_Failure	Binary	No	1.45(1.22, 1.72)	<.001
	Elixahauser_Liver_disease	Binary	No	1.51(1.24, 1.83)	<.001
	Elixahauser_Renal_failure	Binary	No	1.67(1.41, 1.97)	<.001
Preoperative	GFR_Non_African_American	Continuous	N/A	0.97(0.97, 0.98)	<.001
	Blood_Urea_Nitrogen	Continuous	N/A	1.01(1.01, 1.02)	<.001
	Hemoglobin	Continuous	N/A	0.88(0.86, 0.91)	<.001
	International_Normal_Ratio	Continuous	N/A	1.44(1.19, 1.74)	<.001
	CCS_Nephrectomy_partial_or_complete	Binary	No	18.42(14.15, 23.98)	<.001
	CCS_Other_OR_therapeutic_procedures_of_urinary_tract	Binary	No	3.3(2.36, 4.62)	<.001
	CCS_Other_organ_transplantation	Binary	No	5.11(3.68, 7.09)	<.001
	High Risk Surgery	Binary	No	1.81(1.63, 2)	<.001
	missing_Bilirubin_Indirect	Binary	No	0.8(0.71, 0.9)	0.003
	ASA Physical Status	Continuous	N/A	1.54(1.42, 1.67)	<.001

© 2019 Lei VJ et al. *JAMA Network Open.*

eTable 6. **Logistic Regression with Elastic Net Selection Estimates using Perioperative Data**

The final model for the logistic regression with elastic net selection using perioperative data (which includes prehospitalization, preoperative, and intraoperative data) consisted of 22 variables.

**Variable Group**	**Variable Name**	**Type**	**Reference**	**Odds Ratio (95% CI)**	**p-value**
	Intercept	N/A	N/A	0.11(0.06, 0.2)	<.001
Prehospitalization	Age	Continuous	N/A	1.01(1.01, 1.02)	<.001
	Insurance: MEDICAID	Categorical	COMMERCIAL	1.35(1.16, 1.57)	<.001
	Insurance: MEDICARE	Categorical	COMMERCIAL	1.14(1.01, 1.28)	0.036
	Insurance: OTHER	Categorical	COMMERCIAL	2.2(1.65, 2.91)	<.001
	Elixahauser_Congestive_Heart_Failure	Binary	No	1.49(1.25, 1.77)	<.001
	Elixahauser_Liver_disease	Binary	No	1.47(1.2, 1.79)	<.001
	Elixahauser_Renal_failure	Binary	No	1.76(1.48, 2.08)	<.001
Preoperative	GFR_Non_African_American	Continuous	N/A	0.97(0.97, 0.98)	<.001
	Blood_Urea_Nitrogen	Continuous	N/A	1.01(1.01, 1.02)	<.001
	Hemoglobin	Continuous	N/A	0.89(0.87, 0.92)	<.001
	International_Normal_Ratio	Continuous	N/A	1.41(1.17, 1.71)	<.001
	CCS_Nephrectomy_partial_or_complete	Binary	No	13.11(10.09, 17.04)	<.001
	CCS_Other_organ_transplantation	Binary	No	3.66(2.5, 5.35)	<.001
	High Risk Surgery	Binary	No	1.58(1.42, 1.76)	<.001
	missing_Bilirubin_Indirect	Binary	No	0.82(0.72, 0.93)	0.002
	ASA Physical Status	Continuous	N/A	1.49(1.37, 1.62)	<.001
Intraoperative	Time_to_surgery_min	Continuous	N/A	1(1, 1)	0.002
	max_Estimated_Blood_Loss_mL	Continuous	N/A	1(1, 1)	<.001
	max_Crystalloids_mL	Continuous	N/A	1(1, 1)	<.001
	Fresh_Frozen_Plasma_YN	Binary	No	0.97(0.71, 1.32)	0.835
	Platlets_YN	Binary	No	1.06(0.76, 1.48)	0.737
	CaCl_YN	Binary	No	1.62(1.3, 2.03)	<.001

© 2019 Lei VJ et al. *JAMA Network Open.*

eTable 7. Feature Importance for Random Forest and Gradient Boosting Machine Models
Below are the top 10 features ranked by Gini importance for the random forest and gradient boosting machine models.

**Model (# of variables)**	**Top 10 Gini Importance Features**	**Variable Group**	**Gini coefficient**
Random forest -	Age	Prehospitalization	0.176
Prehospitalization (35 variables)	Elixhauser_Renal_failure	Prehospitalization	0.110
	Insurance_commerical	Prehospitalization	0.067
	Elixhauser_Liver_disease	Prehospitalization	0.060
	Insurance_medicare	Prehospitalization	0.049
	Elixhauser_Congestive_Heart_Failure	Prehospitalization	0.048
	Elixhauser_Solid_tumor_without_metastasis	Prehospitalization	0.045
	Elixhauser_Fluid_and_electrolyte_disorders	Prehospitalization	0.043
	Elixhauser_Hypertension_complicated	Prehospitalization	0.040
	Insurance_other	Prehospitalization	0.039
Random forest -	Creatinine_Level	Preoperative	0.087
Preoperative (309 variables)	ASA_Physical_Status	Preoperative	0.083
	GFR_Non_African_American	Preoperative	0.068
	CCS_Nephrectomy_partial_or_complete	Preoperative	0.060
	Age	Prehospitalization	0.060
	Hemoglobin	Preoperative	0.054
	Red_Blood_Cell	Preoperative	0.045
	Blood_Urea_Nitrogen	Preoperative	0.038
	White_Blood_Cell	Preoperative	0.035
	Glucose_Level	Preoperative	0.033
Random forest -	ASA_Physical_Status	Preoperative	0.067
Perioperative (339 variables)	Creatinine_Level	Preoperative	0.067
	GFR_Non_African_American	Preoperative	0.057
	CCS_Nephrectomy_partial_or_complete	Preoperative	0.046
	Hemoglobin	Preoperative	0.037
	Age	Prehospitalization	0.035
	max_Crystalloids_mL	Intraoperative	0.032
	Red_Blood_Cell	Preoperative	0.025
	max_Estimated_Blood_Loss_mL	Intraoperative	0.025
	Surgery_duration_min	Intraoperative	0.025
Gradient Boosting Machine -	Age	Prehospitalization	0.188
Prehospitalization (35 variables)	Elixhauser_Renal_failure	Prehospitalization	0.091
	Insurance_other	Prehospitalization	0.051
	Elixhauser_Liver_disease	Prehospitalization	0.050
	Elixhauser_Congestive_Heart_Failure	Prehospitalization	0.045
	Elixhauser_Solid_tumor_without_metastasis	Prehospitalization	0.040
	Elixhauser_Fluid_and_electrolyte_disorders	Prehospitalization	0.038
	Sex_Female	Prehospitalization	0.030
	Insurance_commerical	Prehospitalization	0.028
	Elixhauser_Chronic_pulmonary_disease	Prehospitalization	0.027
Gradient Boosting Machine -	Creatinine_Level	Preoperative	0.083
Preoperative (309 variables)	CCS_Nephrectomy_partial_or_complete	Preoperative	0.046
	Age	Prehospitalization	0.044
	Prothrombin_Time	Preoperative	0.037
	Hemoglobin	Preoperative	0.036
	Chloride_Level	Preoperative	0.034
	Red_Blood_Cell	Preoperative	0.031
	Glucose_Level	Preoperative	0.029
	White_Blood_Cell	Preoperative	0.029
	Sodium_Level	Preoperative	0.029
Gradient Boosting Machine -	Creatinine_Level	Perioperative	0.075

© 2019 Lei VJ et al. *JAMA Network Open.*

Perioperative (339 variables)	CCS_104	Preoperative	0.038
	max_Estimated_Blood_Loss_mL	Intraoperative	0.033
	Age	Prehospitalization	0.032
	Surgery_duration_min	Intraoperative	0.026
	Max_Urine_Output	Intraoperative	0.023
	mean_Systolic_Blood_Pressure	Intraoperative	0.021
	mean_Diastolic_Blood_Pressure	Intraoperative	0.021
	Hemoglobin	Preoperative	0.020
	White_Blood_Cell	Preoperative	0.020

© 2019 Lei VJ et al. *JAMA Network Open.*

eTable 8. Super Learner Model Performance for Acute Kidney Injury

There were no meaningful differences in model performance when using an ensembling model technique like Super Learner[2] (eTable 3 vs. eTable 8).

Variables	Algorithm	Super Learner Model Weight	Derivation Set, AUC (95% CI)	Validation Set, AUC (95% CI)	Test Set, AUC (95% CI)
**Prehospitalization**	Super Learner Ensemble		0.675 (0.663, 0.686)	0.647 (0.628, 0.667)	0.651 (0.632, 0.671)
	Random Forest	0.040	0.695 (0.684, 0.707)	0.567 (0.546, 0.587)	0.603 (0.583, 0.622)
	XGBoost	0.134	0.713 (0.702, 0.722)	0.619 (0.600, 0.638)	0.644 (0.626, 0.664)
	Penalized Logistic Regression	0.574	0.647 (0.635, 0.658)	0.647 (0.628, 0.666)	0.646 (0.628, 0.664)
	Gradient Boosting Machine	0.252	0.658 (0.647, 0.668)	0.643 (0.622, 0.662)	0.639 (0.619, 0.658)
**Preoperative**	Super Learner Ensemble		0.988 (0.987, 0.989)	0.803 (0.785, 0.818)	0.805 (0.789, 0.820)
	Random Forest	0.318	1.000 (1.000, 1.000)	0.783 (0.765, 0.800)	0.792 (0.776, 0.808)
	XGBoost	0.154	0.971 (0.968, 0.974)	0.779 (0.763, 0.796)	0.780 (0.763, 0.797)
	Penalized Logistic Regression	0.526	0.787 (0.777, 0.797)	0.797 (0.781, 0.813)	0.793 (0.777, 0.809)
	Gradient Boosting Machine	0.002	0.781 (0.772, 0.790)	0.785 (0.767, 0.802)	0.788 (0.772, 0.805)
**Perioperative**	Super Learner Ensemble		0.988 (0.987, 0.989)	0.816 (0.801, 0.830)	0.822 (0.806, 0.838)
	Random Forest	0.249	1.000 (1.000, 1.000)	0.803 (0.788, 0.819)	0.812 (0.797, 0.827)
	XGBoost	0.208	0.984 (0.982, 0.986)	0.786 (0.770, 0.803)	0.796 (0.779, 0.811)
	Penalized Logistic Regression	0.401	0.805 (0.797, 0.814)	0.812 (0.796, 0.827)	0.810 (0.794, 0.825)
	Gradient Boosting Machine	0.142	0.802 (0.792, 0.811)	0.801 (0.785, 0.817)	0.806 (0.791, 0.822)

© 2019 Lei VJ et al. *JAMA Network Open.*

eTable 9. Model Performance for Acute Kidney Injury When Setting Extreme Covariate Values to Missing

There were no meaningful differences in model performance when setting variable outlier data to missing (eTable 3 vs. eTable 9).

**Models**	**Derivation Set (n = 25,616), AUC (95% CI)**	**Validation Set (n = 8,505), AUC (95% CI)**	**Test Set (n = 8,494), AUC (95% CI)**
Logistic Regression with Elastic Net Selection			
Prehospitalization	0.695 (0.685, 0.706)	0.706 (0.687, 0.725)	0.700 (0.681, 0.719)
Preoperative	0.765 (0.756, 0.775)	0.779 (0.762, 0.796)	0.782 (0.765, 0.799)
Perioperative	0.769 (0.759, 0.779)	0.775 (0.758, 0.792)	0.783 (0.766, 0.800)
Random Forest			
Prehospitalization	0.740 (0.730, 0.750)	0.712 (0.693, 0.730)	0.710 (0.692, 0.728)
Preoperative	0.924 (0.920, 0.928)	0.788 (0.771, 0.805)	0.787 (0.770, 0.803)
Perioperative	0.966 (0.964, 0.968)	0.799 (0.783, 0.815)	0.799 (0.782, 0.814)
Gradient Boosting Machine			
Prehospitalization	0.725 (0.714, 0.735)	0.713 (0.694, 0.731)	0.712 (0.693, 0.731)
Preoperative	0.819 (0.809, 0.827)	0.799 (0.783, 0.814)	0.803 (0.788, 0.819)
Perioperative	0.862 (0.854, 0.869)	0.808 (0.792, 0.824)	0.815 (0.801, 0.830)

© 2019 Lei VJ et al. *JAMA Network Open.*

eTable 10. Model Performance in Top 3 Surgical Subgroups in Test Dataset

In response to a reviewer comment, we examined specialty-specific models (top 3 highest volume) and did find some variability, though lower sample sizes seemed like the most important reason for variation in model performance.

	**All Encounters (n = 8,494)**		**Orthopedic Surgery (n = 3,110)**		**General Surgery (n = 1,758)**		**Neurological Surgery (n = 1,267)**	
**Models**	**AKI Rate**	**AUC, (95% CI)**	**AKI Rate**	**AUC, (95% CI)**	**AKI Rate**	**AUC, (95% CI)**	**AKI Rate**	**AUC, (95% CI)**
**Logistic Regression with Elastic Net Selection**								
Prehospitalization	0.099	0.700 (0.681, 0.719)	0.072	0.732 (0.697, 0.767)	0.108	0.660 (0.620, 0.700)	0.052	0.585 (0.514, 0.656)
Preoperative		0.782 (0.765, 0.799)		0.767 (0.733, 0.801)		0.747 (0.711, 0.783)		0.710 (0.642, 0.779)
Perioperative		0.790 (0.773, 0.807)		0.767 (0.734, 0.800)		0.773 (0.739, 0.807)		0.692 (0.621, 0.763)
**Random Forest**								
Prehospitalization	0.099	0.710 (0.691, 0.728)	0.072	0.739 (0.702, 0.771)	0.108	0.657 (0.617, 0.697)	0.052	0.625 (0.562, 0.692)
Preoperative		0.787 (0.769, 0.803)		0.782 (0.750, 0.812)		0.739 (0.706, 0.773)		0.702 (0.630, 0.771)
Perioperative		0.808 (0.792, 0.824)		0.796 (0.754, 0.816)		0.772 (0.737, 0.804)		0.699 (0.626, 0.765)
**Gradient Boosting Machine Models**								
Prehospitalization	0.099	0.712 (0.695, 0.731)	0.072	0.741 (0.706, 0.777)	0.108	0.626 (0.617, 0.698)	0.052	0.624 (0.550, 0.692)
Preoperative		0.804 (0.788, 0.819)		0.786 (0.755, 0.815)		0.745 (0.709,0.777)		0.685 (0.610, 0.753)
Perioperative		0.817 (0.802, 0.832)		0.786 (0.754, 0.816)		0.775 (0.741, 0.808)		0.722 (0.659, 0.780)

Abbreviations: AKI, acute kidney injury; AUC, area under the receiver operating characteristic curve; CI, confidence interval

© 2019 Lei VJ et al. *JAMA Network Open.*

eTable 11. Model Results by Acute Kidney Injury Definition and Modeling Approach

When replicating models for the RIFLE and AKIN AKI definitions, the improvement in model performance across variable groups was similar to performance using the KDIGO definition. The table below shows model performance within the test data set (n = 8,494).

	**Logistic Regression with Elastic Net Selection (n = 8,494)**		**Random Forest (n = 8,494)**		**Gradient Boosting Machine (n = 8,494)**	
**Acute Kidney Injury Definition and Datasets**	**AUC (95% CI)**	**p-value for AUC comparison**	**AUC (95% CI)**	**p-value for AUC comparison**	**AUC (95% CI)**	**p-value for AUC comparison**
KDIGO Acute Kidney Injury						
Prehospitalization variables	0.700 (0.681, 0.719)		0.710 (0.690, 0.728)		0.712 (0.694, 0.731)	
Preoperative variables	0.782 (0.765, 0.799)	<.001[a]	0.787 (0.770, 0.803)	<0.001[a]	0.804 (0.788, 0.819)	<0.001[a]
Perioperative variables	0.790 (0.773, 0.807)	0.023[b]	0.808 (0.790, 0.823)	<0.001[b]	0.817 (0.802, 0.832)	<0.001[b]
RIFLE Acute Kidney Injury						
Prehospitalization variables	0.669 (0.649, 0.688)		0.675 (0.656, 0.693)		0.676 (0.657, 0.692)	
Preoperative variables	0.748 (0.73, 0.766)	<.001[a]	0.757 (0.740, 0.775)	<0.001[a]	0.776 (0.758, 0.791)	0.001[a]
Perioperative variables	0.757 (0.739, 0.775)	0.014[b]	0.775 (0.759, 0.791)	<0.001[b]	0.792 (0.776, 0.808)	<0.001[b]
AKIN Acute Kidney Injury						
Prehospitalization variables	0.698 (0.679, 0.717)		0.707 (0.688, 0.725)		0.711 (0.692, 0.730)	
Preoperative variables	0.782 (0.765, 0.799)	<.001[a]	0.788 (0.772, 0.805)	<0.001[a]	0.802 (0.786, 0.817)	<0.001[a]
Perioperative variables	0.791 (0.774, 0.808)	0.026[b]	0.808 (0.793, 0.823)	<0.001[b]	0.819 (0.804, 0.834)	<0.001[b]

Abbreviations: AUC, area under the receiver operating characteristic curve; CI, confidence interval; KDIGO, Kidney Disease: Improve Global Outcomes; RIFLE, Risk, Injury, Failure, Loss of kidney function, End stage kidney disease; AKIN, Acute Kidney Injury Network

[a]AUC comparison between prehospitalization and preoperative variables; [b]AUC comparison between preoperative and perioperative variables

Area under the receiver operating characteristic curves (AUC) for each model and acute kidney injury definition are shown for the test set. The AUC, or C-statistic, is calculated along with 95% confidence intervals. DeLong's test is used to test for significant difference between model AUCs after adding additional clinical variables ($p < 0.001$).

© 2019 Lei VJ et al. *JAMA Network Open.*

eTable 12. Acute Kidney Injury Risk Stratification Using Alternate Definitions of High Risk in Test Dataset – Rates of Clinical Outcomes by Variable Group

Because the top 20% was an arbitrary cutoff, we also conducted sensitivity analyses of risk stratification using alternate definitions of High Risk at top 10% and top 30%.

Gradient Boosting Machine Acute Kidney Injury Model Risk Stratification	Sample (n = 8,494)	Acute Kidney Injury, No. (%) (n = 845)	In-patient Dialysis, No. (%) (n = 32)	Postoperative Length of Stay ≥7 days, No. (%) (n = 1,669)	In-Hospital Death, No. (%) (n = 58)
**High Risk defined as the Top 10% of predicted risk. Low Risk defined as the Bottom 90% of predicted risk.**					
Prehospitalization variables					
High Risk	850	247 (29.06)	17 (2)	297 (34.94)	19 (2.24)
Low Risk	7,644	598 (7.82)	15 (0.2)	1,372 (17.95)	39 (0.51)
Preoperative variables					
High Risk	850	347 (40.82)	26 (3.06)	421 (49.53)	29 (3.41)
Low Risk	7,644	498 (6.51)	6 (0.08)	1,248 (16.33)	29 (0.38)
Perioperative variables					
High Risk	850	355 (41.76)	28 (3.29)	448 (52.71)	34 (4)
Low Risk	7,644	490 (6.41)	4 (0.05)	1,221 (15.97)	24 (0.31)
**High Risk defined as the Top 20% of predicted risk. Low Risk defined as the Bottom 80% of predicted risk.**					
Prehospitalization variables					
High Risk	1,699	378 (22.25)	22 (1.29)	567 (33.37)	34 (2)
Low Risk	6,795	467 (6.87)	10 (0.15)	1,102 (16.22)	24 (0.35)
Preoperative variables					
High Risk	1,699	495 (29.13)	28 (1.65)	738 (43.44)	40 (2.35)
Low Risk	6,795	350 (5.15)	4 (0.06)	931 (13.7)	18 (0.26)
Perioperative variables					
High Risk	1,699	510 (30.02)	30 (1.77)	774 (45.56)	51 (3)
Low Risk	6,795	335 (4.93)	2 (0.03)	895 (13.17)	7 (0.1)
**High Risk defined as the Top 30% of predicted risk. Low Risk defined as the Bottom 70% of predicted risk.**					
Prehospitalization variables					
High Risk	2,548	485 (19.03)	24 (0.94)	799 (31.36)	36 (1.41)
Low Risk	5,946	360 (6.05)	8 (0.13)	870 (14.63)	22 (0.37)
Preoperative variables					
High Risk	2,548	595 (23.35)	31 (1.22)	990 (38.85)	51 (2)
Low Risk	5,946	250 (4.2)	1 (0.02)	679 (11.42)	7 (0.12)
Perioperative variables					
High Risk	2,548	606 (23.78)	31 (1.22)	1,002 (39.32)	52 (2.04)
Low Risk	5,946	239 (4.02)	1 (0.02)	667 (11.22)	6 (0.1)

Abbreviations: GBM, Gradient Boosting Machines; AKI: acute kidney injury

Risk stratification of gradient boosting machines models in the test set (n = 8,494) using alternate definitions of High Risk. Incidence rates of primary and secondary clinical outcomes were calculated from sample totals. In-patient dialysis was defined using ICD-CM-9 procedure codes (eMethods).

© 2019 Lei VJ et al. *JAMA Network Open.*

eReferences

1. Volume 2: ESRD Analytical Methods. *American Journal of Kidney Diseases.* 2018;71(3):S545-S610.
2. van der Laan MJ, Polley EC, Hubbard AE. Super learner. *Stat Appl Genet Mol Biol.* 2007;6:Article25.
3. Bellomo R, Ronco C, Kellum JA, Mehta RL, Palevsky P, Acute Dialysis Quality Initiative w. Acute renal failure - definition, outcome measures, animal models, fluid therapy and information technology needs: the Second International Consensus Conference of the Acute Dialysis Quality Initiative (ADQI) Group. *Crit Care.* 2004;8(4):R204-212.
4. Mehta RL, Kellum JA, Shah SV, et al. Acute Kidney Injury Network: report of an initiative to improve outcomes in acute kidney injury. *Crit Care.* 2007;11(2):R31.
5. Levey AS, Stevens LA, Schmid CH, et al. A new equation to estimate glomerular filtration rate. *Ann Intern Med.* 2009;150(9):604-612.
6. Quan H, Sundararajan V, Halfon P, et al. Coding algorithms for defining comorbidities in ICD-9-CM and ICD-10 administrative data. *Medical care.* 2005;43(11):1130-1139.
7. Clinical Classifications Software (CCS) for CPT. 2017.
8. Schwarze ML, Barnato AE, Rathouz PJ, et al. Development of a list of high-risk operations for patients 65 years and older. *JAMA Surg.* 2015;150(4):325-331.

© 2019 Lei VJ et al. *JAMA Network Open.*
